# Supplementary material for: Rapid turnover of life-cycle-related genes in the brown algae
Source: Genome Biol. 2019 Feb 14;20:35. doi: 10.1186/s13059-019-1630-6 (PMC6374913; doi:10.1186/s13059-019-1630-6)
Supplement: Supplementary file 3 — Protein alignments, corresponding Gblocks html file and CDS sequences for the lineage conserved gametophyte-biased genes. (ZIP 2109 kb) [file 13059_2019_1630_MOESM3_ESM.zip › Con_GA_alignments/Ec-10_004900.aa_ali.fasta-gb.htm]

Ec-10\_004900.aa.fasta.trans.aa\_ali.fasta


## Gblocks 0.91b Results

Processed file: **Ec-10\_004900.aa.fasta.trans.aa\_ali.fasta**  
Number of sequences: **2**  
Alignment assumed to be: **Protein**  
New number of positions: **193** (selected positions are underlined in blue)

```
                         10        20        30        40        50        60
                 =========+=========+=========+=========+=========+=========+
Ec-10_004900     MAKTPPRARSRSPSPTRWPNNPRAAGGTSAIDRTMEGQTTPEVDEDFLVREWAFTTSEVS
MSTRG.54378.1    ------------------------------------------------------------
                                                                             


                         70        80        90       100       110       120
                 =========+=========+=========+=========+=========+=========+
Ec-10_004900     IQQGEEGVLGRGSYGEVRVATWRGVRVAAKQLHALSGAEAAASLSIEQTAAVRRDLLKEM
MSTRG.54378.1    ------------------------------------------------------------
                                                                             


                        130       140       150       160       170       180
                 =========+=========+=========+=========+=========+=========+
Ec-10_004900     SLLASLRHPNLLLFLGVTYDPHTHNPRSIITELMPSSVYDLLETRGVRLEQHEVLDLASD
MSTRG.54378.1    ------------------------------------------------------------
                                                                             


                        190       200       210       220       230       240
                 =========+=========+=========+=========+=========+=========+
Ec-10_004900     IASGLVYIHGQSPAIVHRDLSSRNVLYDGRTAKLADLGQAKAMGVASAVGGSRQTAMPGA
MSTRG.54378.1    ------------------------------------------------------------
                                                                             


                        250       260       270       280       290       300
                 =========+=========+=========+=========+=========+=========+
Ec-10_004900     MVYAAPEVLTGRYSSSIDVFSFGVLLAQLITGEYPRLDTRKEQVAEAGDRFSLLKPLLDR
MSTRG.54378.1    ------------------------------------------------------------
                                                                             


                        310       320       330       340       350       360
                 =========+=========+=========+=========+=========+=========+
Ec-10_004900     CLSLHAEDRPAAAEALRALEALRADPCAYHPAGHGAGILADRWFQEEQKEKTRGLEARVR
MSTRG.54378.1    ------------------------------------------------------------
                                                                             


                        370       380       390       400       410       420
                 =========+=========+=========+=========+=========+=========+
Ec-10_004900     VHERRLSAEVGRWKEEADRADEMSGKIRAAESERDAAVTRQEELQHSLAVSLAALEEERS
MSTRG.54378.1    ------------------------------------------------------------
                                                                             


                        430       440       450       460       470       480
                 =========+=========+=========+=========+=========+=========+
Ec-10_004900     ARQEIEDQLKEEGEKLRYLQDATERLQEDLLRAQRAVKSAETLQGLAEGNNTLANRAAQE
MSTRG.54378.1    ------------------------------------------------------------
                                                                             


                        490       500       510       520       530       540
                 =========+=========+=========+=========+=========+=========+
Ec-10_004900     AKEARVTAGQRAEQAEAQLAGQVAATREAEARVQQAISRWEGEKVDRLRFRKEYITKCSE
MSTRG.54378.1    -------------------AGQAAATREAEARLRQALSRWEGEKIDRLKYRKEYMAKCNE
                                    #########################################


                        550       560       570       580       590       600
                 =========+=========+=========+=========+=========+=========+
Ec-10_004900     AQERERTVRDLRARLKEATDRLSKYDGLPESQQIRQRMLDLEGDRQRANDETSEREKELE
MSTRG.54378.1    AQERERTVRDLRASLKEAADRLSTYDGLPESQQIRQRMLDLEDDRQRAKDETTEREKELE
                 ############################################################


                        610       620       630       640       650       660
                 =========+=========+=========+=========+=========+=========+
Ec-10_004900     ERQRDIDALNDKLKAVQDARDAALKQSEVLETTVDSVLRDRGDAVKRAEKAGKALTKARK
MSTRG.54378.1    ERQKDVGCLTDKLKAVEDERNLAREHGKTLEEETKDARRDRSDAVQKAEKARKVADKAQR
                 ############################################################


                        670       680       690       700       710       720
                 =========+=========+=========+=========+=========+=========+
Ec-10_004900     QADEDGMQIETLKNEVLGVRKEMKRVKKDAPWVFKGGGKSQGSSSNNTNAADRIRESSNS
MSTRG.54378.1    QAEEDKLQIEKLKDEIAGARKEMKRVRKDAPWL---------------------------
                 ################################                            


                        730       740       750       760       770       780
                 =========+=========+=========+=========+=========+=========+
Ec-10_004900     TSGDTGEIHEQGDGGILGARRRPASAVSSGSGSSDDEEGRSENGAETMVGKAAETGPRGV
MSTRG.54378.1    ------------------------------------------------------------
                                                                             


                        790       800       810       820       830       840
                 =========+=========+=========+=========+=========+=========+
Ec-10_004900     AELLRRHPKRHELQRRGLRALRDLAYKGDEERAAVAAAGGVAAAVHAMNRFPADSRVQVG
MSTRG.54378.1    ------------------------------------------------------------
                                                                             


                        850       860       870       880       890       900
                 =========+=========+=========+=========+=========+=========+
Ec-10_004900     GVKTLAHLAFGNDVNRIEVGSSGGTAAILRTMAANPADPELQGEACTAFTNLSHNCDRNR
MSTRG.54378.1    ------------------------------------------------------------
                                                                             


                        910       920       930       940       950       960
                 =========+=========+=========+=========+=========+=========+
Ec-10_004900     KLVVEGGGLILILNAMQTFPGHPKLQRQACWALLTLAANDEISRVIASEGGVGAIIAAMI
MSTRG.54378.1    ------------------------------------------------------------
                                                                             


                        970       980       990      1000      1010      1020
                 =========+=========+=========+=========+=========+=========+
Ec-10_004900     NNADDTSVQHFGCWALANVGWGQADVQRFAREEGAIEVIQTAVQRFPEHQALVAKARLAS
MSTRG.54378.1    ------------------------------------------------------------
                                                                             


                       1030
                 =========+==
Ec-10_004900     SIILEGVGLAPV
MSTRG.54378.1    ------------
```

```
Parameters used
Minimum Number Of Sequences For A Conserved Position: 2
Minimum Number Of Sequences For A Flanking Position: 2
Maximum Number Of Contiguous Nonconserved Positions: 8
Minimum Length Of A Block: 10
Allowed Gap Positions: None
Use Similarity Matrices: Yes
```

```
Flank positions of the 1 selected block(s)
Flanks: [500  692]  

New number of positions in Ec-10_004900.aa.fasta.trans.aa_ali.fasta-gb:  193  (18% of the original 1032 positions)
```
